# Abnormal Helminth Egg Development, Strange Morphology, and the Identification of Intestinal Helminth Infections

**DOI:** 10.3201/eid2408.180560

**Published:** 2018-08

**Authors:** Sarah G.H. Sapp, Michael J. Yabsley, Richard S. Bradbury

**Affiliations:** University of Georgia, Athens, Georgia, USA (S.G.H. Sapp, M.J. Yabsley);; Centers for Diseases Control and Prevention, Atlanta, Georgia, USA (R.S. Bradbury)

**Keywords:** helminths, morphology, Ascarididae, Schistosomatidae, diagnostics, egg development, nematodes, trematodes, parasites

## Abstract

Occasionally, abnormal forms of parasitic helminth eggs are detected during routine diagnostics. This finding can prove problematic in diagnosis because morphologic analysis based on tightly defined measurements is the primary method used to identify the infecting species and molecular confirmation of species is not always feasible. We describe instances of malformed nematode eggs (primarily from members of the superfamily Ascaridoidea) from human clinical practice and experimental trials on animals. On the basis of our observations and historical literature, we propose that unusual development and morphology of nematode and trematode eggs are associated with early infection. Further observational studies and experimentation are needed to identify additional factors that might cause abnormalities in egg morphology and production. Abnormal egg morphology can be observed early in the course of infection and can confound accurate diagnosis of intestinal helminthiases.

Despite recent advances in molecular diagnostics, microscopic analysis of ova and larvae, and to a lesser degree adult worms, remains the mainstay for the diagnosis of intestinal helminths in humans and animals worldwide. In most cases, such morphologic diagnosis relies upon the identification of the helminth genera or species based on the characteristic morphology of eggs because adult parasites are rarely available. When unfamiliar egg morphologies are observed, parasitologists will often consult with atlases and textbooks that describe the morphology of eggs produced by various species of helminth infecting the host feces being examined to determine the species of helminth concerned. These references generally describe the standard presentation of eggs without consideration of potential abnormal forms. In addition, students are generally only provided the best specimens during practical classes in which they are taught to identify parasites.

The authors of this article conduct disparate work in intestinal helminths of humans and animals. They routinely undertake classical morphologic diagnosis of parasites in their respective roles and have extensive experience in morphologic methodologies. Casual discussions between the authors revealed that they had each observed highly abnormal forms of helminth eggs from humans and animals during the course of their work.

One author (R.S.B.) had observed multiple highly abnormal forms of *Ascaris lumbricoides* roundworms being passed by humans during the course of human intestinal helminth surveillance studies in the eastern Solomon Islands. These forms were passed by different persons and included eggs with double morulae, giant eggs (size ranging up to 110 µm in length), and eggs not conforming to the traditional symmetric, ovoid morphology associated with those of *A. lumbricoides*. Nevertheless, these eggs demonstrated several distinct features that identified them as belonging to *A. lumbricoides*, and they were observed in association with other eggs of *A. lumbricoides* that demonstrated standard morphologic features (Figure 1). All of the eggs were observed in Kato Katz preparations, a method that is known to cause some malformation in helminth eggs, particularly those of schistosomes and hookworms, which will collapse or dissolve, respectively, if the smear is allowed to clear for too long (*1*). However, the degree of morphologic abnormality observed in these specimens was far beyond the relatively minor swelling and clearing of *A. lumbricoides* eggs common to Kato Katz preparations and only occurred occasionally, eliminating artifact of Kato Katz preparation as a cause. The observations of these highly abnormal egg morphologies were made in the context of a population with very high prevalence and intensity of ascariasis (prevalence 53%, with 28% having moderate- to heavy-intensity egg counts) and the possibility that this feature was caused by crowding of gravid female worms in the gut of the host was considered. This author had also previously observed abnormal variations in the eggs of *Schistosoma haematobium*, all recovered from the same urine specimen of a refugee from Africa attending the Royal Hobart Hospital in Tasmania, Australia (Figure 2).

**Figure 1:**
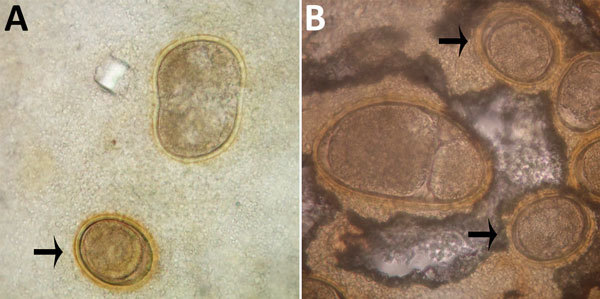
Abnormalities of *Ascaris lumbricoides* eggs from patients in the Solomon Islands, visualized on Kato-Katz. A) Giant egg with irregular indented shape. B) Giant egg with 2 morulae. Arrows indicate eggs of normal morphology. Original magnification ×400.

**Figure 2:**
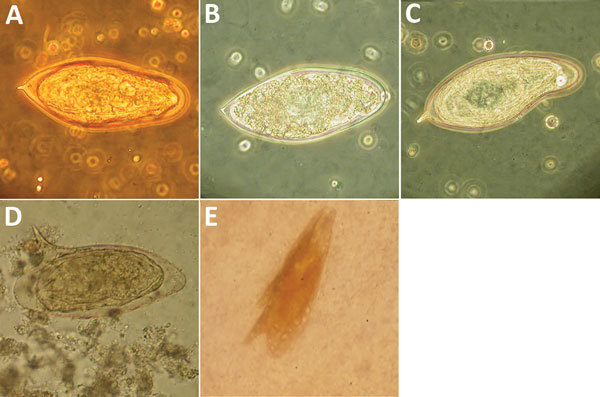
Abnormalities of *Schistosoma* spp. eggs visualized in wet preparation. A) Normal *S. haematobium* egg (≈150 µm long). B) *S. haematobium* egg with reduced terminal spine. C) *S. haematobium* egg with irregular, indented shape. D) Normal *S. mansoni* egg (≈150 µm long). E) *S. mansoni* egg with 2 lateral spines. Original magnification ×400. Panel E image courtesy of John Goldsmid, University of Tasmania (Hobart, Tasmania, Australia).

Other authors (S.G.H.S. and M.J.Y.) made similar observations in regard to ascarid infections of raccoons (*Procyon lotor*) and domestic dogs (*Canis familiaris*). During experimental infections of naive, ascarid-free dogs and captive-bred raccoons with *Baylisascaris procyonis*, the raccoon roundworm, abnormal eggs were observed in fecal flotation exams conducted during the first few days and weeks of patency. Abnormalities observed included eggshell distortions resulting in irregular, crescent, budded, and triangular shapes, and twin eggs conjoined by an eggshell but with separate morulae and vitelline membranes (Figure 3). Many eggs during early patency were unusually oblong but remained within normal size variation. These abnormal eggs were detected in dogs that were infected through ingestion of tissues from infected mice (larvae) and in raccoons that were infected through ingestion of eggs or larvae. Among the 6 raccoons inoculated with larvae, all had a proportion of markedly malformed eggs early in patency; this malformation was also observed in 3 of 4 raccoon-inoculated eggs that became patent. Obviously malformed eggs represented ≈5% of eggs observed after fecal flotation of samples obtained within the first 2 weeks of patency, with limited variation (range 1.5%–7%). The frequency at which these malformed eggs were observed decreased with the length of infection; some animals ceased to pass any malformed eggs after ≈30 days postpatency.

**Figure 3:**
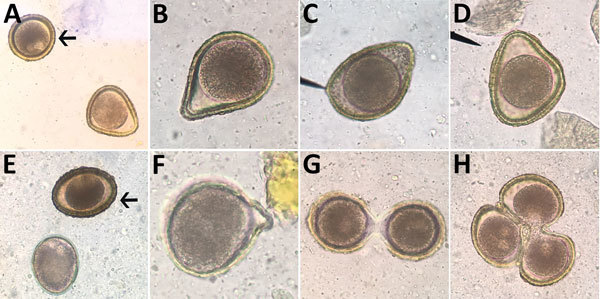
Abnormalities of *Baylisascaris procyonis* eggs shed by experimentally inoculated dogs and raccoons, visualized on fecal flotation. A) Triangular egg. B) Pear-shaped egg. C) Almond-shaped egg. D) Triangular egg with indented edge. E) “Immature” egg with underdeveloped morula and no cortex or proteinaceous coat. F) Budded egg. G) Twin conjoined eggs with separate morulae and vitelline membranes. H) Triplet conjoined eggs with distinct morulae; vitelline membrane might be shared between 2 eggs. Arrows indicate eggs of normal morphology (65–75 µm). Original magnification ×400.

Even for experienced morphologists, variability in size and shape of helminth eggs adds a layer of complexity to diagnosis. For example, unusually large *Trichuris* spp. eggs were observed in the stool of a child in the Bahamas (*2*). The size of these eggs was outside of the typically observed range for human-infecting *T. trichiura* whipworms (normal eggs also were observed in the patient) and instead were within the range of the canine *T. vulpis* and feline *T. campanula* (*felis*) worms. Otherwise, however, the eggs were not morphologically consistent with either of these animal whipworm species. Whether this case was an abnormal egg shedding by *T. trichium* worms, a zoonotic *Trichuris* species, or even human infection with a novel species is unknown. Modern molecular diagnosis would aid in species resolution, but this approach is not necessarily an option in underserved areas.

Morphologic deviation from typical ranges is also an important consideration and source of confounding in studies on natural infections of wildlife. Two authors of this article (S.G.H.S. and M.J.Y.) were consulted by a veterinarians who had received a diagnosis of *Baylisascaris* spp. infection infor a captive bobcat (*Felis rufus*) kitten by a veterinary reference laboratory. The veterinarian requested confirmation because this case would have represented the first report of *Baylisascaris* spp. infecting a feline host. Upon examination, we found the eggs to be mostly morphologically consistent with *Toxocara cati* (size ≈75 × ≈80 µm, generally round to pear shaped, golden in color, and with a finely pitted shell), but some did resemble *Baylisascaris* spp. (smaller size, ≈68 × ≈60 µm, slightly ellipsoid, with a darker, amber color, and with a thicker shell). These eggs were allowed to embryonate for 3 weeks and were then artificially hatched to examine the morphology of larvae. The larvae from the normal and abnormal eggs were morphologically similar to each other and were identified as belonging to the species *T. cati* (≈350 µm long, ≈20 µm midbody diameter; slightly flared anterior end suggesting early development of cervical alae). Because this infection was identified in a kitten, it might represent an additional example of malformed eggs passed early in patency. No follow-up samples or recovered adult worms were available because the host had been treated for the infection.

We conducted a review of the literature to investigate previous descriptions of abnormalities in the morphology of intestinal helminth eggs, with particular reference to ascarids. Matuda summarized the findings of early investigators discussing abnormal forms of *A. lumbricoides* (Technical Appendix) (*3*). These descriptions included several similar to those observed by 1 author (R.S.B.) in the Solomon Islands, which included double morulae in a single egg (categorized by Matuda as category B.I [online Technical Appendix]), enlarged eggs (B.II.a), and deformities of the egg shells (B.III). These observations in *A. lumbricoides* eggs were also similar to deformities observed in the related parasite *B. procyonis* by S.G.H.S. and M.J.Y., including a budded shell (B.III.a), triangular shape (B.III.c), almond to crescent shape (B.III.b), and fused eggs (B.I.a1, B.I.a2, and B.I.b.). No further work was completed on the mechanisms that caused the *Ascaris* egg abnormalities described by Matuda. Similar abnormalities, including united eggs, angular deformities, and unusual position or absence of polar globules, were later observed in structural studies on the poultry ascarids *Ascaridia galli* and *Heterakis gallinae* (*4*). Descriptions of abnormal egg morphology in the literature for other nematode taxa are scarce and perhaps underrecognized. One example found was that of conjoined *Trichuris vulpis* eggs from a routine dog fecal examination (Figure 4).

**Figure 4:**
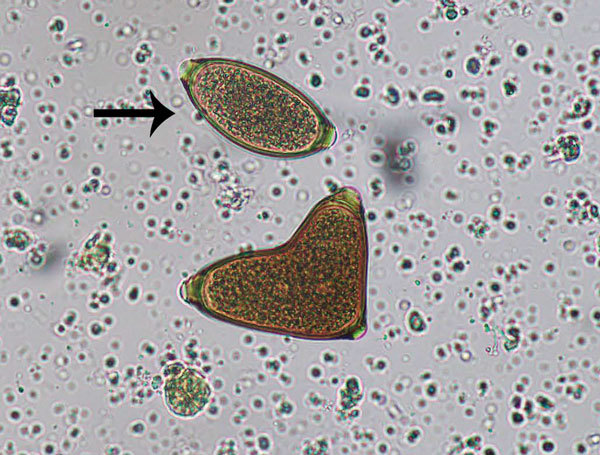
Conjoined *Trichuris vulpis* eggs from a domestic dog, visualized on fecal flotation. Arrow indicates a morphologically normal egg. Original magnification ×400. Photograph by Danielle E. Preston and courtesy of Mani Lejeune, both of the Cornell University College of Veterinary Medicine (Ithaca, NY, USA).

Leiper described variations in the morphology and position of spines of schistosome eggs in Egypt in the first decade of the 20th century (*5*). These abnormal findings led to controversy among investigators of the day regarding the number of species of schistosomes in Egypt. Much later, Goldsmid reported morphologic abnormalities in *Schistosoma mansoni* eggs in Zimbabwe, including a double-spined egg (Figure 2, panel E) (*6*).

The etiology of malformation in these abnormally shaped eggs is unknown. During *B. procyonis* infection trials by S.G.H.S. and M.J.Y., malformed eggs were initially detected in infected dogs, suggesting that the passage of these eggs by dogs might be the result of an abnormal host–parasite relationship, host immunity, or both. Previous and current studies suggest that dogs are poor definitive hosts for *B. procyonis* (*7*; S.G.H. Sapp and M.J. Yabsley, unpub. data) compared with the natural raccoon host. However, the observation of deformed eggs in our subsequent trials with experimentally infected raccoons suggests a predominantly parasite-mediated (as opposed to host-mediated) process. Although not investigated mechanistically in nematodes to our knowledge, abnormalities in trematode (i.e., *Fasciola hepatica* and *Dicrocoelium dendriticum*) egg production have been attributed to differential vitelline gland activity in immature and senescent flukes (*8*). Also, Leiper attributed the observed malformations and variability in spines and shape in eggs produced by *S. haematobium* worms in Egypt to egg production by immature worms (*5*). In 1926, Manter also observed that eggs produced by immature specimens of the fish trematode *Otodistomum cestoides* were undersized and of unusual shape (*9*).

We also considered the possibility that malformed ascarid eggs might be caused by crowding stress on adult worms in high-intensity infections of the gut, given that the *A. lumbricoides* eggs were passed by persons in the Solomon Islands with high-intensity infections (i.e., passing unusually high numbers of eggs). However, the *B. procyonis* infections in the dogs and raccoons were not high-intensity infections; only a moderate egg burden was observed (maximum of ≈600 eggs/g feces in dogs and ≈9,000 eggs/g feces in raccoons). Given the high prevalence and intensity of infection in the Solomon Islands study, the abnormalities seen alongside normally formed eggs could be attributable to an immature female entering patency and producing abnormally shaped eggs alongside several mature females producing normal eggs. Moreover, anecdotal discussions with colleagues in the past have raised the possibility that these abnormalities are attributable to the effects of anthelmintic use on egg development, and sporadic reports exist of malformed eggs after unsuccessful or low-dose sublethal anthelmintic treatment (*10*,*11*). However, none of the hosts that were passing abnormal ascarid eggs during our observations had been treated with anthelmintics in the preceding 3 years. None of the raccoons given piperazine or ivermectin at the conclusion of the study passed abnormal eggs after treatment. Helminth taxa might respond differently to anthelmintics (e.g., ascarid eggs might be less likely to have abnormalities after exposure, whereas this development has been noted in *Trichuris* spp.) (*11*,*12*). Furthermore, some classes of anthelmintics could feasibly interfere with egg development to a stronger degree than others, although data on the subject are limited.

The combined incidental findings described in this report demonstrate the potential for morphologically abnormal helminth egg morphologies to occur in certain host species. These abnormalities might confound or mislead inexperienced morphologists regarding the identity of such helminth eggs and thereby lead to incorrect or missed diagnoses. The current standard morphologic descriptions address only the commonly observed morphologies of intestinal helminth eggs, and the potential for substantial deformity is not generally considered by laboratory staff. In addition, numerous ecologic studies are conducted on parasites of wildlife, which frequently rely on nonlethal sampling through identification of eggs in fecal samples by researchers who might not have extensive training in morphologic identification of parasitic ova. Moreover, many parasite species of wildlife have poorly described or unknown egg morphology data available. The presence of abnormal eggs might lead to spurious descriptions of new species in such studies. Furthermore, many parasite species of wildlife have poorly described or unknown egg morphology data. Should abnormal eggs be observed, especially in an unusual host, molecular characterization might be necessary to obtain a definitive diagnosis.

Our findings and evidence from the historical literature suggests that immaturity of egg-producing helminths might be a major cause of the observed egg deformations we have described. Other etiologic factors such as host immunity cannot be discounted on the basis of such preliminary findings, and more work is needed to understand the causes and mechanisms leading to the passage of deformed eggs. This report serves to highlight the existence of such phenomena and the potentially underrecognized challenge that malformed helminth eggs represent in terms of correctly diagnosing helminth infections in humans and animals. We hope that more research will occur to determine the causes of these aberrations and the diversity of their occurrence across helminth taxa. Such research should consider the intensity of host infection, host or helminth age associations, and, ultimately, immunologic mechanisms. Our collaborative investigation also further highlights how even casual communication between scientists working on very different subjects can be unexpectedly enlightening, particularly in the highly interdisciplinary area of parasitology.

Technical Appendix Matuda classification of abnormal *Ascaris* spp. eggs.
