# Supplementary material for: Abnormal Helminth Egg Development, Strange Morphology, and the Identification of Intestinal Helminth Infections
Source: Emerg Infect Dis. 2018 Aug;24(8):1407–11. doi: 10.3201/eid2408.180560 (PMC6056116; doi:10.3201/eid2408.180560)
Supplement: Technical Appendix — Matuda classification of abnormal Ascaris spp. eggs. [file 18-0560-Techapp-s1.pdf]

Article DOI: https://doi.org/10.3201/eid2408.180560

Abnormal Helminth Egg Development, Strange Morphology, and the Identification of Intestinal Helminth Infections

Technical Appendix

Matuda Classification of Abnormal *Ascaris* spp. Eggs; Adapted from Matuda (1934) (*1*).

A. Normal egg: within typically observed size range and shape.

B. Abnormal egg.

- I. United eggs: this form consists of some eggs covered with the same albuminous membrane.
  - a. Two eggs united.
    - 1. United shells of normal morphology.
    - 2. United shells of abnormal morphology.
    - 3. Two morulae within single shell.
  - b. Three or more eggs united.
- II. Deformity in diameter.
  - a. Abnormally large.
  - b. Abnormally small.
  - c. Giant eggs.
    - 1. Giant eggs with normal shape.
    - 2. Giant eggs with abnormal/irregular shape.
- III. Deformity in shape.
  - a. Budding.
  - b. Indented.
    - 1. Asymmetrical indentation.
    - 2. Symmetrical indentation.
  - c. Angular deformity.
  - d. Abnormally elongate.

Reference

1. Matuda S. Some abnormal eggs of *Ascaris lumbricoides* Linnaeus. Vol Jubil pro Profr Sadao Yoshida. 1939;2:311–4.
